# Multi-Round compared to Real-Time Delphi for consensus in core outcome set (COS) development: a randomised trial

**DOI:** 10.1186/s13063-021-05074-2

**Published:** 2021-02-15

**Authors:** Fiona A. Quirke, Patricia Healy, Elaine Ní Bhraonáin, Mandy Daly, Linda Biesty, Tim Hurley, Karen Walker, Shireen Meher, David M. Haas, Frank H. Bloomfield, Jamie J. Kirkham, Eleanor J. Molloy, Declan Devane

**Affiliations:** 1 Health Research Board Neonatal Encephalopathy PhD Training Network (NEPTuNE), Galway, Ireland; 2 Health Research Board – Trials Methodology Research Network (HRB-TMRN), Galway, Ireland; 3 College of Medicine, Nursing and Health Sciences, National University of Ireland Galway, Galway, Ireland; 4 School of Nursing and Midwifery, National University of Ireland Galway, Galway, Ireland; 5 Family Support Liaison, Irish Neonatal Health Alliance, Wicklow, Ireland; 6 Advocacy and Policymaking, Irish Neonatal Health Alliance, Wicklow, Ireland; 7 Qualitative Research in Trials Centre (QUESTS), National University of Ireland Galway, Galway, Ireland; 8 Paediatrics and Child Health, Trinity College Dublin, Dublin, Ireland; 9 RPA Newborn Care, Sydney Local Health District, Sydney, Australia; 10 Birmingham Women’s and Children’s NHS Foundation Trust, Birmingham, UK; 11 Indiana University School of Medicine Department of Obstetrics and Gynecology, Indianapolis, USA; 12 Liggins Institute, University of Auckland, Auckland, New Zealand; 13 Centre for Biostatistics, University of Manchester, Manchester, UK; 14 Department of Neonatology, Children’s Hospital Ireland at Crumlin and Tallaght, Coombe Women and Infants University Hospital, Dublin, Ireland; 15 Evidence Synthesis Ireland, National University of Ireland Galway, Galway, Ireland; 16 Cochrane Ireland, National University of Ireland Galway, Galway, Ireland

**Keywords:** Real-Time Delphi, Multi-Round Delphi, Core outcome sets, Methodology, Randomised trial

## Abstract

**Background:**

The Delphi method is used in a wide variety of settings as a method of building consensus on important issues. Traditionally, the Delphi method uses multiple rounds of a survey to allow for feedback of other participants’ survey responses in between rounds. By informing participants about how others answer a question or prioritise specific topics, it allows for diverse opinions to inform the consensus process. For this reason, the Delphi method is popular as a consensus building approach in developing core outcome sets (COS), i.e. the minimum agreed set of standardised outcomes that should be measured and reported in studies on a specific health condition. In a COS setting, participants prioritise the importance of outcomes for inclusion in a COS. This usually involves participating in multiple rounds of a survey that can span several weeks or months. Challenges with participant retention have been highlighted in previous COS. We will compare a three-round with a Real-Time Delphi approach on prioritised outcomes. This trial is embedded within the COHESION study which is developing a COS for interventions treating neonatal encephalopathy.

**Methods:**

One hundred and eighty stakeholders (parents/caregivers of infants diagnosed and treated with neonatal encephalopathy, healthcare providers and researchers) will be randomised using stratified randomisation to take part in either the Multi-Round or Real-Time Delphi. Stakeholders will rate the importance of the same set of outcomes in both arms. We will compare the prioritised outcomes at the end of both surveys as well as other parameters such as feedback, initial condition and iteration effects.

**Discussion:**

This trial will provide evidence to inform decisions on the use of Multi-Round compared to Real-Time Delphi survey methods.

**Trial registration:**

NCT04471103. Registered on 14 July 2020.

**Supplementary Information:**

The online version contains supplementary material available at 10.1186/s13063-021-05074-2.

## Introduction

### Background and rationale

The Delphi method is used widely to achieve consensus among experts on a particular topic [1]. It was developed first by Dalkey and Helmer [2] as a way to establish the opinions of experts anonymously on issues related to sensitive military operations. Since then, the Delphi method has transitioned from a pen-to-paper approach to the e-Delphi method, where surveys are carried out online using the Internet with the potential for a global reach and potentially achieving consensus faster than pen-to-paper versions.

The Delphi method, in general, involves multiple stages or rounds of a questionnaire. It is an iterative process. Participants complete an initial survey. When this survey round closes, participants are sent “feedback”. This feedback often includes information such as how the participant answered each question/item on the survey and how each item/question was answered by the participants overall (e.g. the average or median score). Participants are often grouped into subgroups, also referred to as “stakeholder” groups. Each stakeholder group contributes a different expertise based on their connection and experience of the condition being investigated. Responses can be aggregated at the whole group level or at the level of varying stakeholder sub-groups, e.g. patients, healthcare providers and researchers. Participants are given the opportunity to modify how they responded to each question in subsequent rounds with the knowledge of how other participants/participant groups answered the questions. In so doing, it is anticipated that respondents will converge on responses to items within the survey. Each participant remains anonymous to other participants throughout the Delphi process.

The Delphi technique confers several advantages over other methods of reaching consensus such as forums or discussion meetings [3]. Maintaining anonymity by avoiding personal identification of participants from one another is viewed as a strength in achieving consensus, by enabling stakeholders to provide their opinion without the process becoming dominated by more assertive individuals. This idea, of gathering the expertise of a variety of stakeholders to establish consensus on essential outcomes for a particular disease or condition, provides a strong rationale for its use in the development of core outcome sets [4].

A core outcome set (COS) is an agreed minimum set of outcomes or outcome measures. It is a recommendation of “what” should be measured and reported in all trials, other studies and potentially routine clinical practice in a specific area. The Delphi method is used commonly to achieve consensus in COS development [4] where participants rate the importance of outcomes for inclusion in a COS. The process followed is otherwise the same as outlined above.

Concerns have been expressed about the lengthy process of completing multiple Delphi rounds, and time waiting for feedback on consensus results between rounds, as potential causes of lost interest and dissatisfaction among participants [5]. These issues may contribute to challenges in recruitment and retention of participants in this phase of the COS development process.

A “Real-Time Delphi” process may offer benefits in the development of COS. The Real-Time Delphi method was developed by Gordon and Pease [6] to improve the speed and efficiency of gathering opinions of experts and making decisions in situations of urgency. As software has developed since then, so too have approaches to using the Real-Time Delphi method.

The Real-Time Delphi approach maintains the benefits of working toward consensus in a survey setting but does so in a potentially more time-efficient manner. Feedback can be given to participants on the web page in “real-time”. This provides feedback to participants more quickly than at the end of a Multi-Round Delphi survey round, which often last up to 3 weeks in COS [4]. The Real-Time Delphi method comprises a round-less Delphi approach [6], where participants are encouraged to re-visit the survey and re-rate items throughout the period in which the Delphi survey is live. Essentially, it removes the time taken in the Multi-Round Delphi for the survey administrator to evaluate the results and provide feedback to participants. Participants can engage with the consensus process from the outset by seeing how other participants have answered the questions and potentially modifying their answers.

Studies comparing traditional-style Delphi surveys and a “Real-Time” Delphi survey approach include a comparison by Geist [7] of a traditional, Multi-Round, pen-to-paper approach with a Real-Time Delphi model, which found that attrition was lower in the Real-Time Delphi arm. The authors suggested this may have been due to a lack of email prompts engaging participants. This study acknowledged that with improved modelling, including increasing the number of reminders to participants to re-visit the Real-Time Delphi, this approach could be a cost-effective and efficient mode of achieving consensus. Gnatzy et al. [8] conducted a comparison of a traditional, sequential round Delphi and a Real-Time Delphi to see if the survey results were affected by changing the survey method (i.e. using a Real-Time Delphi method). However, although this group describe an in-depth approach of evaluating a Real-Time Delphi model, participants were not randomised, and the surveys took place with participants from different countries and at different times. Any or all of these factors could affect the results of comparing these two Delphi surveys.

Thiebes et al. [9] discuss how the Real-Time Delphi deviates from the characteristics of a Delphi survey as outlined by Rowe, Wright and Bolger [10] who distinguish four key features that characterise a Delphi survey, i.e. “anonymity”, “iteration”, “controlled feedback” and “statistical group response”. “Anonymity” is key within the Delphi process, whereby participants remain anonymous from one another throughout the survey, encouraging all participants to give their opinions without being influenced by more influential individuals. This feature is maintained in the Real-Time Delphi design.

“Iteration”, according to Rowe, Wright and Bolger [10], refers to presenting participants with a questionnaire comprised of multiple rounds and facilitating participants to change how they answer a question. Although the Real-Time Delphi is essentially round-less, it can incorporate iteration by encouraging participants to re-visit and re-answer questions based on changing feedback. This feedback differs from the definition of “Controlled feedback” which Rowe, Wright and Bolger [10] suggest should be given between rounds. Instead, in the Real-Time Delphi, this feedback, such as how the participant answered the question and how other participants, as a group responded to the question, is provided once the participant has submitted their answer. “Statistical group response” refers to the feedback that is provided to the participants. Instead of the feedback being reflective of all participants who have taken part in the survey after each round, the feedback in the Real-Time Delphi is reflective of the participants who have taken part up to that point in the survey. This is why participants are encouraged to re-visit and re-respond to questions if they wish, to get a reflection of the views of participants at different time points and to capture any changes in consensus. Whilst in a Real-Time Delphi, feedback is not “controlled” by limiting feedback only at the end of distinct rounds, it can be argued that the Real-Time Delphi approach still largely incorporates the key features of the Delphi procedure.

Despite claims that a Real-Time Delphi will improve efficiency of the survey process by removing the strict rounds and the time it takes for participants to complete the survey [6, 8], there is no strong evidence on how it compares to the Multi-Round Delphi.

### Objectives

To identify whether different outcomes are prioritised when using a Multi-Round compared with a Real-Time Delphi method in the development of a COS for interventions for the treatment of neonatal encephalopathy.

The unique outcomes identified at the end of both survey arms will be brought forward to consensus meetings. At these meetings, the most important outcomes will be prioritised by key stakeholders (parents/caregivers of infants diagnosed and treated for neonatal encephalopathy, healthcare providers and researchers). An online discussion and final vote will be used to decide on which outcomes should be included in the final COS for the treatment of neonatal encephalopathy.

### Trial design and explanation

**Fig. 1:**
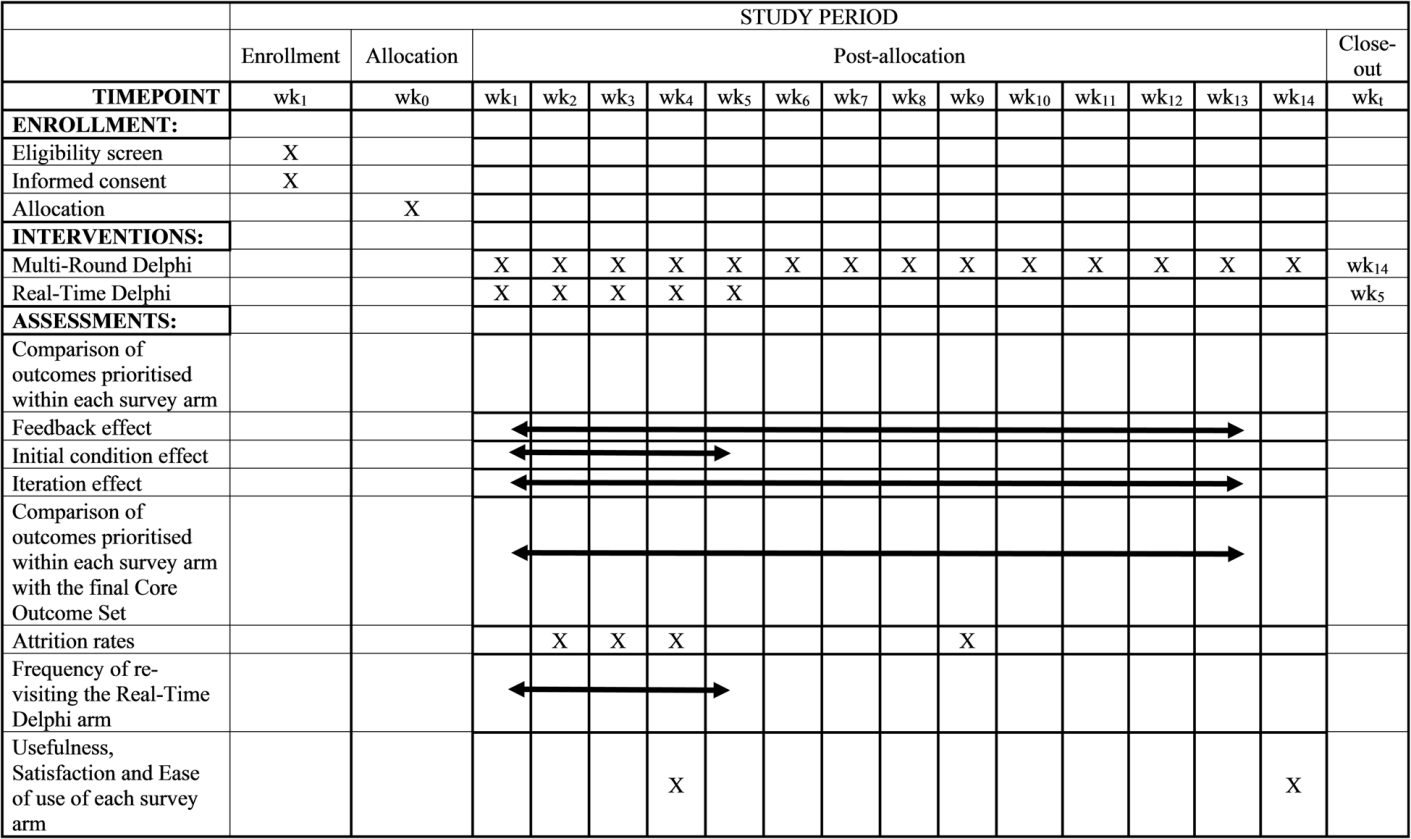
Schedule of enrolment, interventions and assessments for the trial, adapted from the SPIRIT 2013 schematic diagram available at https://www.spirit-statement.org/schedule-of-enrolment-interventions-and-assessments/

This study is a two-group, parallel randomised trial. For this protocol, we have followed the SPIRIT 2013 Checklist [11] (Additional file 1); see also Fig. 1. For more information on the COS, please see the *In-press* protocol that accompanies this paper “COHESION: Core Outcomes in Neonatal Encephalopathy (Protocol)” (Additional file 1).

## Methods

### Study setting

Both the Multi-Round and Real-Time Delphi surveys will take place online using the Calibrum (Surveylet) system, which is General Data Protection Regulation (GDPR) compliant.

### Eligibility criteria

We will recruit parents whose infants have been diagnosed with and received treatment for neonatal encephalopathy or caregivers who may care for the infant, healthcare providers (including neonatal nurse practitioners, midwives, obstetricians, neonatologists/paediatricians, neonatal/paediatric neurologists, general practitioners who provide long-term care for children with neonatal encephalopathy, policymakers and other healthcare providers such as therapists (speech, physical, etc.), etc.), and researchers with expertise in neonatal encephalopathy treatment to take part in the Delphi surveys.

### Interventions

**Fig. 2:**
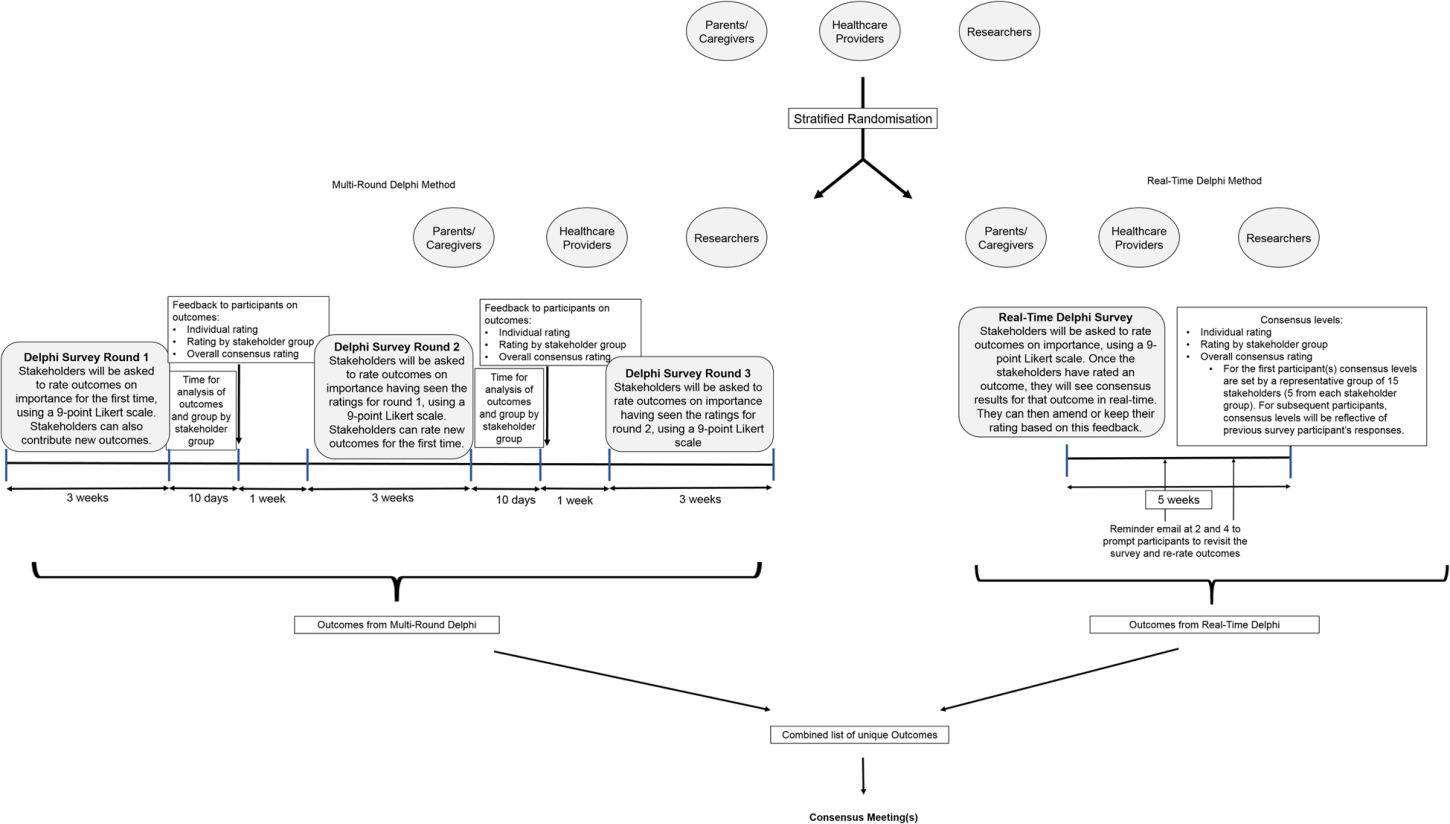
Projected timeline and plan for trial

Respondents will be randomised to a Real-Time Delphi or a three-round e-Delphi (see Fig. 2, projected timeline and plan for trial), with the same outcomes to be rated in each survey.

#### Real-Time Delphi method

The Real-Time Delphi survey will contain outcomes identified from a systematic review of (1) randomised trials and (2) systematic reviews of randomised trials, of interventions for the treatment of neonatal encephalopathy. In addition, outcomes identified as important to parents/caregivers of infants who have been diagnosed with and received treatment for neonatal encephalopathy identified through one-to-one interviews will be included. The survey will also include a short questionnaire seeking participant demographic information.

Participants will be asked to rate the importance of each outcome for inclusion in the COS using a 9-point Likert scale used commonly in the Delphi method for COS development [4]. For each outcome, the participant will be able to view how they rated each outcome, the overall rating results for each outcome and the results for each outcome from each stakeholder group, in real-time. This information will be displayed as feedback immediately after a participant rates an outcome for the first time. With this feedback, participants will be able to modify their answer or keep it the same before moving on to the next outcome. Participants will be encouraged to suggest additional outcomes that they feel are omitted from the list of outcomes presented in the survey. Additional outcomes will be reviewed within 2 days by a representative subgroup from the COHESION Steering Group (i.e. parents/caregivers, healthcare providers and researchers) and included in the Real-Time Delphi survey, if not already captured in the outcomes list. As the degree of consensus changes, depending on how participants rate an outcome, we will encourage participants to re-visit the survey and review their answers based on the degree of consensus. If a participant scores 6 or more points different on the 9-point scale from that given by overall group consensus score (e.g. if the overall group consensus is that an outcome is extremely important and ranks 9 on the Likert scale, and the participant rates it extremely unimportant, as 1 on the Likert scale and vice versa), they will be given the option to comment on the reasoning behind the rating they have given. Participants can save and re-visit the survey to complete the questions and/or modify their answers.

The order in which outcomes will be presented to participants will be randomised within their outcome domains, as Brookes et al. [12] have shown that question order may influence the level of response. In addition, a lay-explanation of outcomes and terminology used in the Delphi survey will be provided as this has been shown to affect the retention of stakeholders positively in a Delphi survey [5].

To avoid a small group of initial participants having a significant influence on the consensus levels and thereby potentially influencing subsequent participants to answer based on those consensus levels, a representative group of stakeholders (5 of each parent/caregivers, healthcare providers and researchers) will vote on the importance of the outcomes before the survey goes live to participants. In this way, consensus information will be available to the first online participant in the same way it will be available for subsequent participants. The timing and frequency of email reminders will be decided by the COHESION Steering Group based on temporal responses.

#### Multi-Round Delphi method

##### First round

Similar to the Real-Time Delphi, the first-round of the Multi-Round Delphi survey will contain outcomes identified from the systematic review and qualitative interviews and will also include a short questionnaire seeking participant demographic information. Participants will rate the importance of each outcome using the same scale and will be invited to contribute new outcomes that they deem to be important, as in the Real-Time Delphi processes above. As in the Real-Time Delphi, participants who vote substantially differently to the overall consensus levels will be invited to provide a reason for their vote.

As described in the Real-Time Delphi above, outcomes will be randomised within domains and lay-explanations will be provided for each outcome. All outcomes will be included in round two of the survey. Participants will be sent email reminders to encourage them to take part in the survey. The timing and frequency of reminders will be decided on by the COHESION Steering Group based on responses over time.

##### Second round

The second-round survey will contain all outcomes from round one. Any new outcomes suggested by participants of the Multi-Round Delphi survey will be reviewed by a subgroup of the COHESION Steering Group, as in the Real-Time Delphi. Unique outcomes in the Multi-Round Delphi will be incorporated into the survey in the second round. Additional outcomes suggested in either survey arm will only be added to that survey arm (i.e. unique outcomes suggested by participants in the Multi-Round Delphi will not be incorporated into the Real-Time Delphi, and vice versa). The rating and consensus results will be based on the consensus criteria outlined in Table 1. Results from round one of the survey will be presented as the proportion of participants rating each outcome on each of the points in the 1 to 9 Likert scale, by each stakeholder group, as well as the overall consensus score. Results will be presented graphically and numerically. Participants will be given the opportunity to re-score the outcomes from round one based on (a) their individual score from the first round of the survey (b) results from each stakeholder group and (c) the consensus results overall for that outcome. In addition, participants will be asked to rate, for the first time, the additional outcomes that may be introduced following round one.

**Table 1:** Consensus classification

Consensus classification	Description	Definition
Consensus in (parent/caregiver-weighted vote)	Consensus that the outcome should be included in the core outcome set	70% or more participants overall scoring as 7 to 9 AND < 15% participants scoring a 1 to 3 OR > 70% or more of parent group scoring as 7 to 9
Consensus out	Consensus that the outcome should not be included in the COS	50% or fewer participants scoring as 7 to 9 in each stakeholder group
Neither consensus in nor consensus out (undetermined consensus)	Uncertainty about the importance of the outcome so retain for next round	Anything else

Once again, participants will be asked to rate the outcomes using a 9-point Likert scale. Participants will also be asked of their willingness and availability to attend the subsequent consensus meetings.

##### Third round

In the third-round survey, participants who completed round two will be presented with outcomes from round two that were rated based on the consensus criteria outlined in Table 1. Participants will be asked to re-rate the retained outcomes using the same 9-point Likert scale. At the end of both surveys, unique outcomes meeting the consensus criteria (Table 1) (i.e. outcomes classified as “consensus in” and neither “consensus in” nor “consensus out”) will be brought for discussion at consensus meeting(s) with a global representative of stakeholders (parents/caregivers, healthcare providers and researchers) to decide on the final COS.

### Recruitment

Purposeful sampling will be used to ensure that those with opinions or known expertise in neonatal encephalopathy in both low-to-middle-income and high-income countries will inform this study. The target sample (parents/caregivers, healthcare providers and researchers with expertise in neonatal encephalopathy) will be accessed through invitational emails, electronic discussion lists, those who have participated in previous work or research in this area and other experts in neonatal encephalopathy who have published on neonatal encephalopathy as identified in the systematic review and qualitative interviews, through professional organisations, and support networks. Each organisation will be sent an email containing an information leaflet and an invitation to participate in COHESION (Additional file 1), which they will be asked to circulate to potential participants using their usual means of communication, i.e. mailing lists, website and social media activities. The email invitation will have an electronic link attached that will enable participants to register to participate and seek their consent. Participants will only be able to start the survey after they have given consent. Participants will be allocated randomly to a traditional, three-round Delphi survey or a Real-Time Delphi survey.

### Randomisation

We will use stratified randomisation of stakeholders (parents/carers, healthcare providers and researchers) on a 1:1 ratio. The randomisation sequence is generated using a computerised random number generator. This is based on the stakeholder’s unique identifier value which is generated by stakeholders selecting which stakeholder category they belong to in an initial demographic survey (i.e. parent, caregiver, neonatal nurse practitioners, midwives, obstetricians, neonatologists/paediatricians, neonatal/paediatric neurologists, general practitioners who provide long-term care for children with neonatal encephalopathy, policymakers and other healthcare providers such as therapists (speech, physical, etc.), etc.), and researchers with expertise in neonatal encephalopathy treatment. Based on their stakeholder category, participants will be grouped into three stakeholder groups (parents/caregivers, healthcare providers and researchers). Once the participant has clicked the survey link, they will be provided with access to the study information and Participant Information Leaflet. They will then be asked to click, within the survey, to provide consent. Demographic information will be collected, and the participant will be randomised within the survey platform. The user is automatically directed to the Multi-Round or Real-Time Delphi survey, based on their random allocation stratified for each of the three stakeholder groups in each survey arm.

Participants in both study arms will be able add comments and additional outcomes and we will compare how often this feature was used in the two arms. We will also ask participants in a short questionnaire, sent by email to those who completed each survey, how they found answering each survey, using a modified USE (Usefulness, Satisfaction and Ease of use) questionnaire [13, 14].

### Outcomes

#### Primary

We will compare the lists of outcomes at the end of both the Real-Time (week 5) and Multi-Round Delphi processes (week 14), and how the outcomes were rated based on the consensus criteria in Table 1.

#### Secondary outcomes

1. We will compare the outcomes that are prioritised (based on Table 1), from each survey arm, against the final COS.

Additional secondary outcomes are informed by previous comparison of Multi-Round Delphi method and the Real-Time Delphi method by Gnatzy et al. [8]:

2. *Feedback effect*

We will calculate the “feedback effect”, which will inform if, and how, the feedback provided to participants resulted in them amending how they rated an outcome. In the Multi-Round Delphi, participants will be given time (1 week) to process the feedback before the next round, where they can re-rate the outcomes. In contrast, in the Real-Time Delphi, participants will be provided with feedback in real-time. They will be given the option to change their rating or keep their answer the same before moving on to the next outcome. To estimate the feedback effect, we will test the null hypothesis:

H0_1_: There is no difference in how participants react to feedback in both survey arms

We will use the following model to estimate this effect:

$$ {cor}_{kl}={b}_o+{b}_1\ast {dev}_{kl}+{b}_2\ast \gamma k+{b}_3\ast \left(\gamma k\ast {dev}_{kl}\right) $$

- *k*: participants of both Multi-Round and RT Delphi surveys
- *l*_s_: the projection dependent on *s* which indicates whether the projections (outcome rating) are from the Real-Time (*s* = 1) or Multi-Round (*s* = 2) Delphi study.
- cor_*kl*_ will indicate how strongly the *expected-probability (EP) values* were corrected after feedback was given.
- Dev_*kl*_: how strongly a stakeholder deviated from the final expected-probability value.
- γk∈{0, 1}: an indicator variable which takes on the value 1 if the expert *k* belongs to the Real-Time and 0 if an expert belongs to the Multi-Round Delphi study.
- *b*_1_: slope coefficient
- *b*_2_: indictor variable
- *b*_3_: interaction term

3. *Initial condition effect*

To ensure that the first participant of the Real-Time Delphi survey is provided with feedback, a representative group of stakeholders (five of each parents/carers, healthcare providers and researchers) will vote on the importance of the outcomes (i.e. “initial conditions”) before the survey goes live to the public. These “initial conditions” may have an impact on how outcomes are rated subsequently during the survey process; we call this the “initial condition” effect. In addition, it may be suggested that participants engaging later in the survey have a greater level of feedback than early participants. This might influence how outcomes are re-rated. To identify if the initial conditions effect the final list of outcomes, we will test the hypothesis:

H0_2_: There is no effect of the initial conditions on the outcomes that emerge as important at the end of the Delphi survey.

We will conduct a regression analysis with initial conditions and final results. The initial conditions will be the independent variable and final results will be the dependent variable. Furthermore, we will test whether the initial conditions cause participants to change their rating on outcomes compared to late participants. For this, we will use a linear regression model.

$$ {\mathrm{cor}}_{ij}={\beta}_0+{\beta}_1\ast {\mathrm{dev}}_{ij}+\beta 2\ast {\upvarphi}_i+{\beta}_3\ast \left({\upvarphi}_i\ast {\mathrm{dev}}_{ij}\right) $$

- *i*: participants in the Real-Time Delphi survey
- *j*: the projection (outcome rating)
- cor_*ij*_: how strongly the stakeholder’s *EP* assessment was altered after proving feedback
- dev_*ij*_: how strongly a stakeholder differed from their *EP* value
- φ_i_ ∈{0,1} represents an indicator variable where the value is 1 if the stakeholder *i* is an early participant (week 1) or 0 if the stakeholder is a late participant (week 4).

This model was developed by Gnatzy et al. [8] predicts how the deviation from average EP impacts corrections whilst accounting for possible differences between early and late respondents.

4. *Iteration effect*

In a Multi-Round Delphi survey, participants can only re-rate the outcomes in the second and third rounds. However, in the Real-Time Delphi, participants have the opportunity to re-visit the survey and re-rate the outcomes as many time as they wish. This difference in the iteration (“iteration effect”) could impact the consensus process.

We will compare any changes in standard deviation (SD) of the EP in both survey arms to test the hypothesis that:

H0_3_: There is no difference in the consensus process between the Real-Time Delphi and the Multi-Round Delphi surveys.

### Sample size

Although there is a lack of evidence-based guidance for the optimal sample size for each group participating in the Delphi survey, we aim to recruit a minimum of 180 participants overall (minimum of 30 per each of three major stakeholder group categories, i.e. parents/caregivers whose infants have been diagnosed with or treated for neonatal encephalopathy, healthcare providers and researchers with expertise in neonatal encephalopathy treatment) for each Delphi survey (i.e. 90 participants in the Multi-Round Delphi survey and 90 participants in the Real-Time Delphi survey) and retain participants using the retention methods as described.

### Data collection methods

This trial involves the use of an online survey (questionnaire) tool using Calibrum (Surveylet).

In the Multi-Round Delphi arm, participants must complete all rounds of the Delphi for their rating to be counted and to influence the outcomes emerging from the Delphi and moving forward to the consensus meeting(s) (Phase 4). In the Real-Time Delphi arm, participants must rate an outcome at least twice for their rating score to be counted. The Delphi surveys will aim to follow the timeline as outlined in Fig. 2. However, the process may be iterative, and timelines may be adjusted.

### Data monitoring

A Data Monitoring Committee is not needed in this study. Steering Group members for COHESION are responsible for trial governance and will monitor outputs continuously from the Delphi processes and pay close attention to retention within the trial and ensure reminder emails are employed to tackle this. Monitoring the data will not influence the participants within their intervention; it will only ensure target sample sizes are achieved.

### Protection of human participants

The COHESION project has received ethical approval from the National University of Ireland, Galway (approval reference: 19-Apr-14). Consent will be obtained from participants and they will retain the right to withdraw from the study at any point. Participants will have up to 1 week to consent but may choose to consent more quickly. We will track participant responses using their name and email addresses. However, all responses will remain confidential and individual names will not be linked directly to individual responses in any reports at any time during or after the study. The web-based system Calibrum (Surveylet) that will be hosting the survey will maintain data behind a firewall accessible only by the researchers who must provide passwords and user-IDs. In addition, all data will be analysed according to groups rather than by individual person.

## Dissemination of findings

Findings from the trial will be communicated to all participants of the trial by email. The results of the trial will be disseminated to stakeholders through channels that were used for recruitment, conference presentations and journal papers.

## Trial status

Protocol version 1 (01/09/20). Participant recruitment has not yet commenced. Recruitment will be completed by 01/09/21.

## Supplementary Information

**Additional file 1: Appendix A.** COHESION Consent form – Delphi survey. **Appendix B.** COHESION Participant Information Leaflet – Delphi Survey. **Appendix C.** Spirit 2013 Checklist.

## Data Availability

All datasets used and/or analysed during the COHESION study will be held by COHESION team as f.quirke1@nuigalway.ie.

## Abbreviations

COS: Core outcome set
COHESION: Core Outcomes in Neonatal Encephalopathy
SPIRIT: Standard Protocol Items: Recommendations for Interventional Trials
USE: Usefulness, Satisfaction, Ease of Use
GDPR: General Data Protection Regulation
SD: Standard deviation
EP: Expected probability
