# Supplementary material for: Multi-Round compared to Real-Time Delphi for consensus in core outcome set (COS) development: a randomised trial
Source: Trials. 2021 Feb 15;22:142. doi: 10.1186/s13063-021-05074-2 (PMC7885346; doi:10.1186/s13063-021-05074-2)
Supplement: Supplementary file 1 — Additional file 1: Appendix A. COHESION Consent form – Delphi survey. Appendix B. COHESION Participant Information Leaflet – Delphi Survey. Appendix C. Spirit 2013 Checklist. [file 13063_2021_5074_MOESM1_ESM.docx]

**Additional file 1**

*Appendix A: COHESION Consent form – Delphi survey*


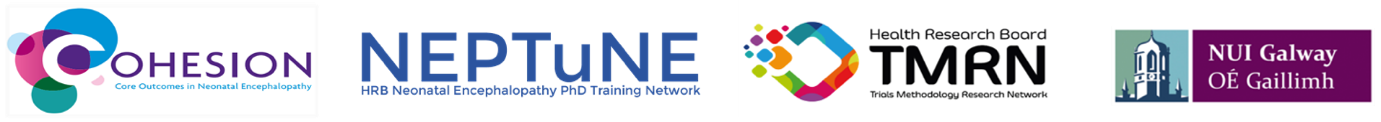


**Delphi Survey Consent Form**

**COHESION (Core Outcomes in Neonatal Encephalopathy)**

1. First/Given name

|  |
| --- |

2. Last/Family name

|  |
| --- |

3. Country (where you currently reside)

|  |
| --- |

4. Email *(To enable us to provide you with a copy of feedback and to send you reminders to participate in this survey)*

|  |
| --- |

5. Please confirm your email address

|  |
| --- |

6. Which of the following groups do you most represent? We know that many people will fulfil more than one role but please select **ONE** category that describes you **best**:

Mother of infant with neonatal encephalopathy

Father of infant with neonatal encephalopathy

Other family Member/Carer of infant with neonatal encephalopathy

Obstetrician

Neonatologist


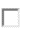
GP


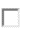
Neurologist

Neonatal Nurse

Midwife

Paediatrician

Policy maker

Researcher/Academic with expertise in neonatal encephalopathy

Other [Please Specify]

**7. Please confirm that you understand the information provided and what taking part in the study will involve.**

- Yes I understand what taking part will involve

**8. Please confirm that you understand that taking part is voluntary and that you are free to withdraw from the study at any time without giving reason;**

- Yes, I understand that participation is voluntary

**9. Please confirm that you understand that the data that you provide will be stored securely at the National University of Ireland Galway for seven years following the completion of the project and understand that members of the study team will have access to this data.**

- Yes, I understand that the data I provide will be securely stored for seven years following the completion of the project

10. Please confirm that you give permission for information collected about you to be stored or electronically processed for the purpose of research and to be used in related studies or other studies in the future but only if the research is approved by a Research Ethics Committee.

**11. If you are happy to take part in this study, please tick "I agree" and then "register" to be taken to first page of the Delphi study.**

 I agree

*Appendix B: COHESION Participant Information Leaflet – Delphi Survey*


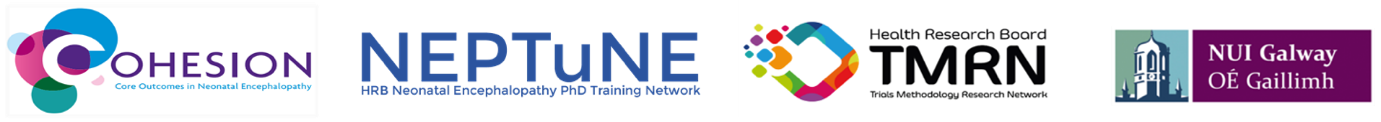


**Participant Information Leaflet**

COHESION (Core Outcomes in Neonatal Encephalopathy)

**Introduction**

We are really pleased that you are interested in taking part in an important research study about neonatal encephalopathy. This Participant Information Leaflet will tell you about this research study, and what taking part means for you.

Please read this leaflet carefully and ask the COHESION research team if you have any questions. We have put the contact details (phone number, email address and postal address) at the end of this leaflet.

Before you decide if you would like to take part, it is important to understand why this research is being done and what it would mean for you.

The aim of the study, called ‘COHESION’, is to develop a core outcome set for use in research studies of treatments for neonatal encephalopathy.

**Why is this study important?**

*Neonatal encephalopathy* is a condition that can occur in newborn infants when they have had a lack of oxygen, and this can cause complications with the infant’s brain. Neonatal Encephalopathy is one of the leading causes of brain injury in newborn infants.

At the moment, different studies into how to best treat neonatal encephalopathy measure how well the treatment works in different ways (called ‘outcomes’). This often means that when health care professionals or parents want to compare or combine different studies, they are unable to do so.

We know it would be impossible to measure everything; so in this study we want to find out which outcomes should be collected in all studies. This set of outcomes is called a ‘core outcome set’.

Please see video <https://www.youtube.com/watch?v=g1MZi2mzK1U> for explanation on core outcome sets.

**What is a core outcome set?**

Core outcomes sets (COS) are the minimum set of outcomes that should be measured and reported in studies evaluating a health intervention. An intervention is anything that aims to make a change to someone’s health. For example, providing a counselling service, giving a drug, or giving people information and training are all described as interventions. Researchers may wish to explore other outcomes relevant to their research, but the COS should always be included. Agreeing the set of outcomes that must be reported in all studies allows us to pool the evidence from different studies, which helps inform health care decisions and also reduces research waste.

**How do I know if I can take part in this study?**

We want to find out what outcomes are most important to (i) parents of infants who have been diagnosed with, and received for neonatal encephalopathy, or other family members who may care for the infant, (ii) healthcare professionals and (iii) researchers or other groups with expertise in neonatal encephalopathy.

You can take part in this study if you are over 18 years of age and are;

- parents, or other family members who may care for the infant or
- healthcare provider or
- Researcher/Academic with expertise in neonatal encephalopathy

**What will I have to do?**

If you agree to take part, you will take part in an online survey called a ‘Delphi survey’ where we will provide a list of possible outcomes related to the treatment of neonatal encephalopathy and ask you to rate the importance of each outcome on a 9-point scale.

When you sign up, you will be allocated by computer (at random) to answer the survey in one of two formats.

Format 1: we will send you a series of 3 online surveys called ‘rounds’ over a 4-6 month period. Each ‘round’ will take approximately 15-20 minutes to complete.

Format 2*:* we will send you a single survey that remains live for a 5-week period. It will take approximately 15-20 minutes to complete the survey. You must visit the survey and rate all the outcomes at least twice during the 5-week period.

The outcomes from Format 1 and Format 2 will be brought to a face-to-face meeting where a final list of outcomes for inclusion in the COS will be agreed

**Are there any benefits or risks to me taking part?**

Your participation will help us establish which outcomes are important and should be measured in all future studies of treatments for neonatal encephalopathy. There are no known risks to taking part in this study.

**Do I have to take part?**

No. You do not have to take part if you don’t want to. You have the right to stop being in the study at any time. If you decide not to take part in this study, or if you decide to stop, you do not have to give a reason.

**Voluntary participation**

Participation is entirely voluntary, and you have the right to withdraw from the study at any time. If you decide not to participate in this study, or if you withdraw, there will be no negative consequences, and you will not be expected to give any reason for your decision. We do hope that you will consider completing all questions within the survey, but if you do decide to exit the study early, all questions that you had completed up to that point will be submitted as complete. If you are a maternity service user and decide not to participate, or to withdraw from the study, your care will not be affected in any way. If you do decide to stop being in the study, please send an email to the COHESION research team to tell us this.

**Confidentiality**

Your identity will remain confidential. All data will be coded, meaning that your name will not be published, and it will not be disclosed. All data retrieved from the survey will be stored securely in the National University of Ireland, Galway under the stewardship of the research team and destroyed after a period of seven years as in accordance with the National University of Ireland, Galway Data Retention Policy.

**What will happen to the findings of this study?**

The findings of the survey will inform the development of a core set of outcomes to determine the effectiveness of treatment for neonatal encephalopathy. The findings of the interviews may be submitted to a journal for publication.

**Compensation**

This study is covered by standard institutional indemnity insurance. Nothing in this document restricts or curtails your rights.

**Funding**

This study has been funded through the Health Research Board (Ireland).

**Has this study received ethical approval?**

Yes, this study has received approval from the following research ethics committee

National University of Ireland, Galway Research Ethics Committee

Research Office

Room 212

Research and Innovation Centre

NUI Galway

Tel: 353 91 495312

**Who can I ask if I have questions?**

You can get more information about the study by asking the COHESION research team. Contact details are as follows:

COHESION (Core Outcomes in Neonatal Encephalopathy) Team

Fiona Quirke, PhD Fellow, NUI Galway ([f.quirke1@nuigalway.ie](mailto:f.quirke1@nuigalway.ie); +353 91 495 481).

Prof. Declan Devane, Professor of Midwifery, NUI Galway [declan.devane@nuigalway.ie](mailto:declan.devane@nuigalway.ie)

**Additional Information**

If you have any issues or queries about the data protection for this project, please find the contact email address of the NUI Galway Data Protection Officer at [dataprotection@nuigalway.ie](mailto:)

Please note that you have the right to request from the data controller for COHESION, access to your data, to rectify or erase your data, or to object to the processing of your data.

Please note that you have the right to lodge a complaint with the Data Protection Commissioner, see [www.dataprotection.ie](http://www.dataprotection.ie) for contact details

Thank you for taking the time to read the information within this participant information leaflet. We hope you will consider taking part.

**If you would like to take part in this survey, please register your interest by clicking on "register" below and filling out the consent form.**

**REGISTER**

*Appendix C: Spirit 2013 Checklist*

SPIRIT 2013 Checklist: Recommended items to address in a clinical trial protocol and related documents*

| Section/item | ItemNo | Description |  |
| --- | --- | --- | --- |
| Administrative information |  |  |  |
| Title | 1 | Descriptive title identifying the study design, population, interventions, and, if applicable, trial acronym | Page 1 |
| Trial registration | 2a | Trial identifier and registry name. If not yet registered, name of intended registry | Page 2, https://clinicaltrials.gov/ct2/show/NCT04471103 |
|  | 2b | All items from the World Health Organization Trial Registration Data Set | Please refer to Item 2a and <https://clinicaltrials.gov/ct2/show/NCT04471103> |
| Protocol version | 3 | Date and version identifier | See Header |
| Funding | 4 | Sources and types of financial, material, and other support | Page 18 |
| Roles and responsibilities | 5a | Names, affiliations, and roles of protocol contributors | Page 18 & 19 |
|  | 5b | Name and contact information for the trial sponsor | Page 18 |
|  | 5c | Role of study sponsor and funders, if any, in study design; collection, management, analysis, and interpretation of data; writing of the report; and the decision to submit the report for publication, including whether they will have ultimate authority over any of these activities | Not applicable, The funding body itself is not involved in the design of the study and collection, analysis, and interpretation of data and in writing the manuscript |
|  | 5d | Composition, roles, and responsibilities of the coordinating centre, steering committee, endpoint adjudication committee, data management team, and other individuals or groups overseeing the trial, if applicable (see Item 21a for data monitoring committee) | Page 16 |
| Introduction |  |  |  |
| Background and rationale | 6a | Description of research question and justification for undertaking the trial, including summary of relevant studies (published and unpublished) examining benefits and harms for each intervention | Page 3 - 6 |
|  | 6b | Explanation for choice of comparators | Page 3-6 |
| Objectives | 7 | Specific objectives or hypotheses | Page 7 |
| Trial design | 8 | Description of trial design including type of trial (eg, parallel group, crossover, factorial, single group), allocation ratio, and framework (eg, superiority, equivalence, noninferiority, exploratory) | Page 7 |
| Methods: Participants, interventions, and outcomes |  |  |  |
| Study setting | 9 | Description of study settings (eg, community clinic, academic hospital) and list of countries where data will be collected. Reference to where list of study sites can be obtained | Page 7 |
| Eligibility criteria | 10 | Inclusion and exclusion criteria for participants. If applicable, eligibility criteria for study centres and individuals who will perform the interventions (eg, surgeons, psychotherapists) | Page 7 & 8 |
| Interventions | 11a | Interventions for each group with sufficient detail to allow replication, including how and when they will be administered | Page 8 - 11 |
|  | 11b | Criteria for discontinuing or modifying allocated interventions for a given trial participant (eg, drug dose change in response to harms, participant request, or improving/worsening disease) | Not applicable, no biological specimens will be collected as part of this trial |
|  | 11c | Strategies to improve adherence to intervention protocols, and any procedures for monitoring adherence (eg, drug tablet return, laboratory tests) | Not applicable, no biological specimens will be collected as part of this trial |
|  | 11d | Relevant concomitant care and interventions that are permitted or prohibited during the trial | Not applicable, this trial will be conducted online |
| Outcomes | 12 | Primary, secondary, and other outcomes, including the specific measurement variable (eg, systolic blood pressure), analysis metric (eg, change from baseline, final value, time to event), method of aggregation (eg, median, proportion), and time point for each outcome. Explanation of the clinical relevance of chosen efficacy and harm outcomes is strongly recommended | Page 13 - 15, For the primary outcome, we will compare the lists of outcomes at the end of both the Real-Time (Week 5) and Multi-Round Delphi processes (Week 14). |
| Participant timeline | 13 | Time schedule of enrolment, interventions (including any run-ins and washouts), assessments, and visits for participants. A schematic diagram is highly recommended (see Figure) | See Figures 1 & 2 |
| Sample size | 14 | Estimated number of participants needed to achieve study objectives and how it was determined, including clinical and statistical assumptions supporting any sample size calculations | Page 15 & 16 |
| Recruitment | 15 | Strategies for achieving adequate participant enrolment to reach target sample size | Page 12 |
| Methods: Assignment of interventions (for controlled trials) |  |  |  |
| Allocation: |  |  |  |
| Sequence generation | 16a | Method of generating the allocation sequence (eg, computer-generated random numbers), and list of any factors for stratification. To reduce predictability of a random sequence, details of any planned restriction (eg, blocking) should be provided in a separate document that is unavailable to those who enrol participants or assign interventions | Page 12 |
| Allocation concealment mechanism | 16b | Mechanism of implementing the allocation sequence (eg, central telephone; sequentially numbered, opaque, sealed envelopes), describing any steps to conceal the sequence until interventions are assigned | Page 12 |
| Implementation | 16c | Who will generate the allocation sequence, who will enrol participants, and who will assign participants to interventions | Page 12 |
| Blinding (masking) | 17a | Who will be blinded after assignment to interventions (eg, trial participants, care providers, outcome assessors, data analysts), and how | Not applicable, blinding will not be carried out |
|  | 17b | If blinded, circumstances under which unblinding is permissible, and procedure for revealing a participant’s allocated intervention during the trial | Not applicable, blinding will not be carried out |
| Methods: Data collection, management, and analysis |  |  |  |
| Data collection methods | 18a | Plans for assessment and collection of outcome, baseline, and other trial data, including any related processes to promote data quality (eg, duplicate measurements, training of assessors) and a description of study instruments (eg, questionnaires, laboratory tests) along with their reliability and validity, if known. Reference to where data collection forms can be found, if not in the protocol | Page 16 |
|  | 18b | Plans to promote participant retention and complete follow-up, including list of any outcome data to be collected for participants who discontinue or deviate from intervention protocols | Page 9 - 11 |
| Data management | 19 | Plans for data entry, coding, security, and storage, including any related processes to promote data quality (eg, double data entry; range checks for data values). Reference to where details of data management procedures can be found, if not in the protocol | Page 16 & 17 |
| Statistical methods | 20a | Statistical methods for analysing primary and secondary outcomes. Reference to where other details of the statistical analysis plan can be found, if not in the protocol | Page 13 - 15 |
|  | 20b | Methods for any additional analyses (eg, subgroup and adjusted analyses) | Page 13 - 15 |
|  | 20c | Definition of analysis population relating to protocol non-adherence (eg, as randomised analysis), and any statistical methods to handle missing data (eg, multiple imputation) | Page 13 - 15 |
| Methods: Monitoring |  |  |  |
| Data monitoring | 21a | Composition of data monitoring committee (DMC); summary of its role and reporting structure; statement of whether it is independent from the sponsor and competing interests; and reference to where further details about its charter can be found, if not in the protocol. Alternatively, an explanation of why a DMC is not needed | Page 16 |
|  | 21b | Description of any interim analyses and stopping guidelines, including who will have access to these interim results and make the final decision to terminate the trial | Page 16 |
| Harms | 22 | Plans for collecting, assessing, reporting, and managing solicited and spontaneously reported adverse events and other unintended effects of trial interventions or trial conduct | Page 16 & 17 |
| Auditing | 23 | Frequency and procedures for auditing trial conduct, if any, and whether the process will be independent from investigators and the sponsor | Not applicable, major auditing of the trial is not anticipated as the trial will be conducted online |
| Ethics and dissemination |  |  |  |
| Research ethics approval | 24 | Plans for seeking research ethics committee/institutional review board (REC/IRB) approval | Page 17 |
| Protocol amendments | 25 | Plans for communicating important protocol modifications (eg, changes to eligibility criteria, outcomes, analyses) to relevant parties (eg, investigators, REC/IRBs, trial participants, trial registries, journals, regulators) | Not applicable, protocol amendments will not directly affect participants of the trial |
| Consent or assent | 26a | Who will obtain informed consent or assent from potential trial participants or authorised surrogates, and how (see Item 32) | Page 17 |
|  | 26b | Additional consent provisions for collection and use of participant data and biological specimens in ancillary studies, if applicable | Not applicable, no biological specimens will be collected as part of this trial |
| Confidentiality | 27 | How personal information about potential and enrolled participants will be collected, shared, and maintained in order to protect confidentiality before, during, and after the trial | Page 16 & 17 |
| Declaration of interests | 28 | Financial and other competing interests for principal investigators for the overall trial and each study site | Page 18 |
| Access to data | 29 | Statement of who will have access to the final trial dataset, and disclosure of contractual agreements that limit such access for investigators | Page 18 |
| Ancillary and post-trial care | 30 | Provisions, if any, for ancillary and post-trial care, and for compensation to those who suffer harm from trial participation | Not applicable, this trial will be conducted online and participation should not cause harm to participants |
| Dissemination policy | 31a | Plans for investigators and sponsor to communicate trial results to participants, healthcare professionals, the public, and other relevant groups (eg, via publication, reporting in results databases, or other data sharing arrangements), including any publication restrictions | Page 17 |
|  | 31b | Authorship eligibility guidelines and any intended use of professional writers | Page 18 |
|  | 31c | Plans, if any, for granting public access to the full protocol, participant-level dataset, and statistical code | Page 17 |
| Appendices |  |  |  |
| Informed consent materials | 32 | Model consent form and other related documentation given to participants and authorised surrogates | See Appendices |
| Biological specimens | 33 | Plans for collection, laboratory evaluation, and storage of biological specimens for genetic or molecular analysis in the current trial and for future use in ancillary studies, if applicable | Not applicable, no biological specimens will be collected as part of this trial |
| *It is strongly recommended that this checklist be read in conjunction with the SPIRIT 2013 Explanation & Elaboration for important clarification on the items. Amendments to the protocol should be tracked and dated. The SPIRIT checklist is copyrighted by the SPIRIT Group under the Creative Commons “Attribution-NonCommercial-NoDerivs 3.0 Unported” license. |  |  |  |
